# Extracorporeal membrane therapy in a case of ruptured abscess on the mitroaortic intervalvular fibrosa associated to multisystem inflammatory syndrome: a case report

**DOI:** 10.1186/s43044-025-00609-3

**Published:** 2025-01-24

**Authors:** Lina P. Montaña-Jimenez, Ana M. Aristizabal, Carlos A. Guzmán-Serrano, Cesar Cely Reyes, Juan Fernando Vélez Moreno, Gastón Castillo

**Affiliations:** 1 Facultad de Ciencias de la Salud, Universidad Icesi, Cali, Colombia; 2 Servicio de Cardiología Pediátrica, Departamento de Materno-Infantil, Fundación Valle del Lili, Cali, Colombia; 3 Centro de Investigaciones Clínicas, Fundación Valle del Lili, Av. Simón Bolívar - Carrera 98 # 18-49, Cali, Colombia; 4 Servicio de Cirugía Cardiaca Pediátrica, Fundación Valle del Lili, Cali, Colombia; 5 Intensivista Pediátrico, Fundación Clínica Infantil Club Noel, Cali, Colombia

**Keywords:** COVID-19, Multisystem inflammatory syndrome, Mitroaortic intervalvular fibrosa, Hemofiltration, Case report

## Abstract

**Background:**

The mitroaortic intervalvular fibrosa is an avascular structure near the left ventricular outflow tract, between the mitral and aortic valves. Mitroaortic intervalvular fibrosa complications, such as tamponade, hemopericardium, and abscesses, are rare and often diagnosed postmortem. On the other hand, the COVID-19 pandemic notably impacted pediatric patients with congenital heart diseases, who frequently presented cardiac complications including arrhythmias, elevated troponins, myocarditis, and heart failure. However, the rupture of the mitroaortic intervalvular fibrosa kept being unusual, making this case a rare presentation of a COVID-19 complication. The objective of this text is to present an infrequent presentation of COVDI-19 complications, and the approach given at our institution which proved to be effective, and further supports the positive findings described in the literature regarding the utility of extracorporeal hemofiltration membranes.

**Case presentation:**

A case of 15-year-old female, without any prior risk factors or cardiac comorbilities, who developed acute myocarditis, linked to COVID-19 Multisystem Inflammatory Syndrome is presented. She deteriorated despite adequate treatment, presenting mitroaortic intervalvular fibrosa rupture, leading to urgent surgical repair and requiring extracorporeal membrane hemofiltration for cytokine removal, therapeutic approach that proved to be effective. Postoperatively, she received intensive care and antibiotics, showing significant cardiac improvement. Noteworthy, hemofiltration was crucial in managing the cytokine storm, contributing to her recovery and subsequent discharge for continued medical management.

**Conclusion:**

An abscess of the mitroaortic intervalvular fibrosa, though rare, represents a significant challenge to clinicians to diagnose. In patients with a history of COVID-19, especially when multisystem inflammatory syndrome is suspected, thorough evaluation is warranted to rule out cardiovascular complications, even in the absence of pre-existing cardiac conditions. This case contributes to our evolving understanding of the cardiovascular implications of COVID-19 and underscores the potential utility of various approaches, including the use of filtration membrane technologies.

## Introduction

COVID-19 is a described infectious disease that has had significant cardiovascular implications, particularly in individuals with congenital heart disease in which it poses a high morbidity and mortality rate [1]. Despite the diagnosis of COVID-19, other diagnoses should not be excluded since its typical presentation, or Multisystem Inflammatory Syndrome (MIS-C) associated to COVID-19, are significant mimickers of associated diseases [1]. Infants are at higher risk among the general population with COVID-19 infection. Common types of cardiovascular complications or sequelae include elevated troponin levels, arrhythmias, myocarditis, and heart failure [1, 2]. However, the rupture of the mitroaortic intervalvular fibrosa (MAIF) has been poorly reported, and no single case, up to our knowledge, has been linked to COVID-19, making this case a rare complication of the disease. The objective of this text is to present an infrequent presentation of COVDI-19 complications, and the approach given at our institution which proved to be effective, and further supports the positive findings described in the literature regarding the utility of extracorporeal hemofiltration membranes.

## Case presentation

A 15-year-old female patient with no significant personal or family medical history presented with an abscess on the right forearm, which resolved on its own, one week before the onset of symptoms. She sought medical attention due to a clinical picture evolving over two days, consisting of fever, general malaise, chest pain associated with coughing, difficulty breathing, and the appearance of bruising lesions on the skin. Positive troponins prompted a referral to a higher level of care with suspicion of myocarditis.

The patient was admitted at a level III hospital with marked respiratory distress, tachycardia, tenderness on palpation of the lower left hemithorax, decreased breath sounds in the left basal region, audible systolic murmur in all areas, grade I edema in the lower limbs. Due to deterioration, she required invasive mechanical ventilation and inotropic and vasoactive support. The initial echocardiogram showed a hyperdynamic left ventricle, mild diastolic dysfunction, preserved systolic function, moderate to severe mitral regurgitation with hemodynamic repercussion, mild left atrial dilatation, and venocapillary pulmonary hypertension. Acute myocarditis and suspicion of MIS-C associated with COVID-19 were considered due to positive IgG results for SARS-CoV-2. She had a poor clinical evolution with multiorgan dysfunction, shock, ventilatory failure, acute kidney injury, and hematological involvement. She also received pentaglobulin as adjunctive management.

The patient's clinical condition did not improve, necessitating increased ventilatory parameters. She showed elevated acute-phase reactants, especially C-reactive protein and D-dimer (see Table 1).

**Table 1:** Laboratory blood tests prior to referral to level IV

Laboratory	Result	Reference values
*SARS-CoV-2*		
Antigen	Negative	Negative
IgG	2.61 (Positive)	Negative
IgM	0.8 (Negative)	Negative
Respiratory Molecular Panel Film Array	Not detectable	Not detectable
C reactive protein	135.2 mg/L	0–0.5 mg/dL
Troponins	1060 ng/ml	*0–20.3 ng/L
Blood culture	*Staphylococcus aureus* MRSA	
*Complete blood tests*		
Total white blood cell count	25.580 × 10^3^/uL	3.98–10.04 × 10^3^/uL
Lymphocytes	1.590 × 10^3^/uL	1.18–3.74 × 10^3^/uL
Neutrophils	23.930 × 10^3^/uL	1.56–6.13 × 10^3^/uL
Monocytes	2.40 × 10^3^/uL	0.24–0.36 × 10^3^/uL
Hemoglobin	9.4 g/dl	11.2–15.7 g/dl
Hematocrit	28.6%	34.1–44.9%
Mean corpuscular volume	82.2 fl	79.4–94.8 fl
Platelets	170.000 × 10^3^/uL	182–369 × 10^3^/uL

*Troponin reference values are standardized for patients over 20 years, with no established values for children in our laboratory

Due to deterioration, Ceftaroline and Caspofungin were used, and a new echocardiographic control was requested, where a rupture of the left Valsalva sinus with a shunt toward the left atrium was suspected, associated with severe aortic insufficiency, moderate to severe mitral insufficiency with hemodynamic repercussion, and severe left atrial dilation. Given the severity of the findings, an urgent referral to a level IV institution for emergent cardiothoracic management was made.

At the present institution, and she was evaluated by an inpatient consul of pediatric infectious diseases, who recommended continuing antibiotic treatment with vancomycin and adding gentamicin. A new echocardiogram was performed, revealing a ruptured abscess involving the MAIF and the left coronary sinus, global pericardial effusion with some echocardiographic signs of cardiac tamponade with a trivalvular aortic valve with mild insufficiency, compromise of the left coronary artery, and mild mitral insufficiency. Given the echocardiographic findings and the patient's clinical condition, an urgent surgical intervention was decided. Additionally, it was decided to place a 12 Fr, 13 cm Mahurkar femoral catheter and to initiate continuous veno-venous hemofiltration with oXiris® hemofilter for filtration, cytokine removal, endotoxin removal, local antiseptics, and anticoagulant treatment. The patient also received immunoglobulin enriched with IgG, IgM, and IgA.

Surgical findings described an abscess at the MAIF involving the left coronary valve at its nadir and the anterior leaflet of the mitral valve in the A3 segment with excrescences. Valvuloplasty was performed successfully. The cardiopulmonary bypass time was 180 min, and the clamp time was 156 min. A mediastinal VAC was left in place and removed the next day.

In the intensive care unit, inotropic and ventilatory support was continued for 24 h, as well as diuretics and the previously described antibiotic management, completing four weeks of vancomycin and two weeks of gentamicin according to the pediatric infectious diseases’ recommendation. A control echocardiogram was performed on the first postoperative day, reporting a patch on the MAIF positioned and aortic plasty, mild to moderate systodiastolic dysfunction of the left ventricle without signs of pulmonary hypertension, mild aortic and mitral insufficiency, no other residual defects, left pleural effusion, or residual thrombi or vegetations; inotropic dilator therapy was initiated based on these findings. The inotropic dilator was discontinued after two days due to clinical improvement, and Staphylococcus aureus was isolated in the vegetation culture. Finally, she was transferred to the ward for continued antibiotic management due to promising clinical progress. On the sixth postoperative day, the chest tube was removed due to minimal production. Due to favorable progress, she was referred for continued medical management.

The respective informed consent and authorization for publication of the case and images was duly completed by the patient’s legal guardian. Approval from ethics committee was obtained prior to the submission of the main text.

## Discussion

**Fig. 1:**
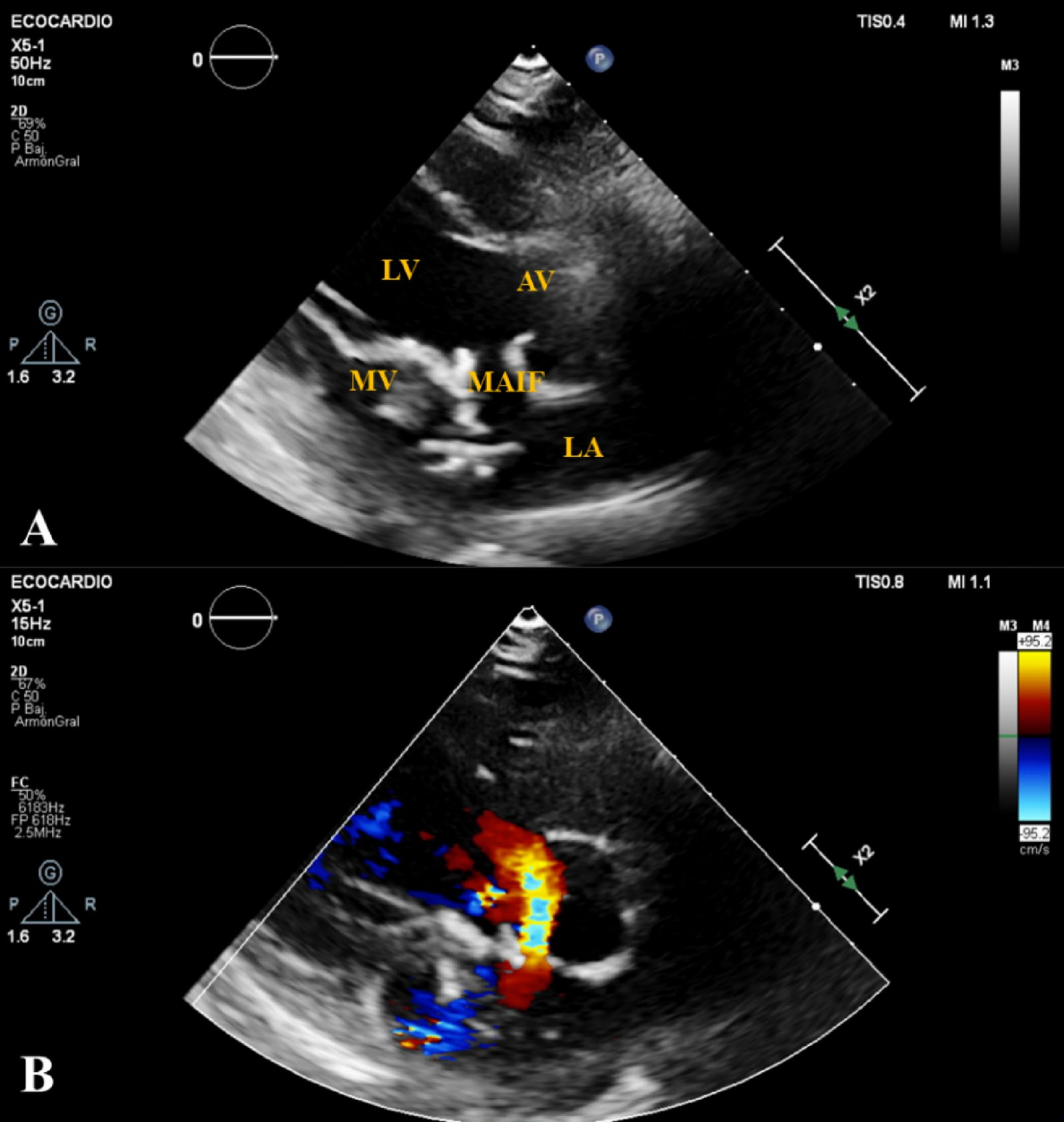
**A** An aberrant communication between the mitral valve and the aortic valve, corresponding to the rupture of the MAIF, is observed. **B** An abnormal flow through the MAIF is detected using color Doppler echocardiography. *LA* Left atrium, *MV* Mitral Valve, *LV* Left Ventricle, *AV* Aortic Valve

The MAIF refers to an avascular structure positioned adjacent to the left ventricular outflow tract, between the mitral valve and the aortic valve. Cardiac tamponed, hemopericardium, fistulae into the adjacent cardiac chamber, arrhythmias, and myocarditis, are some of the potentially life-threatening repercussions [3, 4]. Furthermore, the occurrence of abscesses within this region, is rare and has been underdiagnosed in numerous cases, or have been identified primarily postmortem [4, 5]. Although echocardiography could be used to diagnose the disease, due to its variety of symptoms it is possible that such exam is not routinely done. In this case, echocardiography served as the sole imaging modality required to establish the differential diagnosis and guide the decision-making process, with surgical findings subsequently confirming the suspected diagnosis (Fig. 1).

The expansion of the abscess around the aorta is a progressive phenomenon where initial deep tissue inflammation results in thickening of the MAIF, from there advancing to the development of an abscess and subsequently leading to the formation of a pseudoaneurysm [4]. This is an infrequent presentation of infective endocarditis, usually associated to a previous cardiac infective focus, bacteremia or sepsis [5]. Potential risk factors include structural abnormalities, cardiac tumors, intracardiac devices, venous catheters, prolonged hospitalization, extended courses of antibiotic therapy, among other factors. [3, 4, 6]. These in turn, can precipitate the formation of pseudoaneurysms and subsequent complications [4]. Hence, pseudoaneurysms of the MAIF and intracardiac masses are primary considerations of differential diagnosis [4, 6]. However, none of the risk factors were present in the described patient.

Regarding COVID-19, the infection by SARS-CoV2 entails various pathophysiological pathways, encompassing ACE2 receptor upregulation in individuals with pre-existing cardiovascular conditions, ACE2 downregulation during the infectious phase which exacerbates the loss of the protective effects mediated by the ACE2/Ang1-7 axis and intensifies the detrimental effects of AngII/AT1 axis, the persistence of NETs expression and activity systemically, cellular stress and induction of cellular senescence, direct endothelial cell injury, and indirect endothelial damage via oxidative stress, among other factors [7]. The engagement on the cardiovascular system produces myocardial injury due to a severe acute inflammatory response, as seen in cytokine storms, viral invasion of cardiomyocytes resulting in cellular damage, and ischemic injury in the presence of severe hypoxia due to acute lung injury [1, 7]. Due to the presence of inflammation and an excessive production of cytokines, MIS-C in children has been related to COVID-19 infection [7, 8]. It tends to appear 2–4 weeks after the infection with SARS-CoV2. Although the precise mechanisms underlying the genesis of MIS-C and its cardiac manifestations are unknown, although a possible delayed cellular and humoral immune origin has been suggested [8]. For instance, myocardial injury may present on patients with MIS-C, stemming from acute myocarditis or ischemia secondary to coronary artery involvement [8]. However, this is more widely seen in patients with prior cardiovascular disease. Since the patient presented fever, markedly elevated inflammatory markers, evidence of multiorgan involvement as well as elevated troponins and findings in echocardiography, MIS-C was diagnosed following the Royal College of Paediatrics and Child Health’s criteria [8].

On the other hand, the approach for MIS-C has evolved over the course of the pandemic, with immunoglobulins and steroids emerging as beneficial interventions [8]. In severe cases, adjunctive therapies such as hemofiltration employing extracorporeal cytokine removal devices have been proposed as innovative approaches, considering the involvement of cytokines in the disease process [8]. While such interventions have demonstrated efficacy in adults with septic shock and other immune-mediated disorders, the majority of supporting evidence for MIS-C derives from case reports illustrating their effectiveness in patient management [9–11].

In the present case, a 4-in-1 filter utilizing extracorporeal blood purification was employed, consisting in a layer of polyethyleneimine (a positively charged molecule) that can adsorb negatively charged molecules such as endotoxins and was used preloaded with 4500 IU/m^2^ of heparin. While most available devices target a single entity, such as endotoxins or cytokines, the membrane in use serves four purposes, combining properties to remove cytokines and endotoxins, provide renal replacement function, and offer anti-thrombotic properties. However, it should be noted that the efficacy of these membranes is still a matter of debate, requiring further multicenter randomized studies to establish positive or meaningful results [9–11]. These membranes are designed to reduce cytokine concentrations below toxic levels, thus preventing their harmful effects. Additionally, they aim to eliminate pathogen-associated molecular patterns to mitigate an excessive immune response. Moreover, these membranes may modulate immune processes by removing leukocytes or reprogramming cellular functions.

## Conclusions

An abscess of the MAIF, though rare, represents a significant challenge to clinicians to diagnose. In patients with a history of COVID-19, especially when MIS-C is suspected, thorough evaluation is warranted to rule out cardiovascular complications, even in the absence of pre-existing cardiac conditions. This case contributes to our evolving understanding of the cardiovascular implications of COVID-19 and underscores the potential utility of various approaches, including the use of filtration membrane technologies.

## Data Availability

Information regarding informed consent, clinical history or any other required data will be sent to the journal in case it is required.

## Abbreviations

MAIF: Mitroaortic intervalvular fibrosa
MIS-C: Multisystem inflammatory syndrome
